# TOPK/PBK is phosphorylated by ERK2 at serine 32, promotes tumorigenesis and is involved in sorafenib resistance in RCC

**DOI:** 10.1038/s41419-022-04909-3

**Published:** 2022-05-11

**Authors:** Huimin Sun, Jianzhong Zheng, Juanjuan Xiao, Juntao Yue, Zhiyuan Shi, Zuodong Xuan, Chen Chen, Yue Zhao, Wenbin Tang, Shaopei Ye, Jinxin Li, Qiumin Deng, Lei Zhang, Feng Zhu, Chen Shao

**Affiliations:** 1 Central Laboratory, Xiang’an Hospital of Xiamen University, Xiamen, 361102 Fujian China; 2 The Key Laboratory for Endocrine-Related Cancer precision Medicine of Xiamen, Xiamen, 361102 Fujian China; 3 School of Medicine, Xiamen University, Xiamen, 361102 Fujian China; 4 Department of Urology, Xiang’an Hospital of Xiamen University, Xiamen, 361102 Fujian China; 5 Cancer Research Institute, the Affiliated Hospital of Guilin Medical University, Guilin, 541001 Guangxi China; 6 Guangxi Health Commission Key Laboratory of Novel Onco-Kinases in Target Therapy, the Affiliated Hospital of Guilin Medical University, Guilin, 541001 Guangxi China; 7 Department of Urology, 985th hospital of PLA, Taiyuan, 030002 Shanxi China; 8 Department of Public healthy, Xiamen University, Xiamen, 361102 Fujian China; 9 Guangxi Key Laboratory of Molecular Medicine in Liver Injury and Repair, the Affiliated Hospital of Guilin Medical University, Guilin, 541001 Guangxi China

**Keywords:** Predictive markers, Renal cancer

## Abstract

TOPK/PBK (T-LAK Cell-Originated Protein Kinase) is a serine/threonine kinase that is highly expressed in a variety of human tumors and is associated with poor prognosis in many types of human malignancies. Its activation mechanism is not yet fully understood. A bidirectional signal transduced between TOPK and ERK2 (extracellular signal-regulated kinase 2) has been reported, with ERK2 able to phosphorylate TOPK at the Thr9 residue. However, mutated TOPK at Thr9 cannot repress cellular transformation. In the present study, Ser32 was revealed to be a novel phosphorylated site on TOPK that could be activated by ERK2. Phospho-TOPK (S32) was found to be involved in the resistance of renal cell carcinoma (RCC) to sorafenib. Herein, combined a TOPK inhibitor with sorafenib could promoted the apoptosis of sorafenib-resistant RCC. High expression of HGF/c-met contributes to activation of p-TOPK (S32) during the development of sorafenib resistance in RCC. The current research presents a possible mechanism of sorafenib resistance in RCC and identifies a potential diagnostic marker for predicting sorafenib resistance in RCC, providing a valuable supplement for the clinically targeted treatment of advanced RCC.

## Introduction

RCC account for 2.7% of adult malignancies [1, 2]. Approximately 10% of RCC patients are diagnosed with advanced RCC, and 30% of patients are diagnosed with metastatic RCC (mRCC) [3, 4]. The 5-year survival rate of mRCC patients is less than 12% [5–7]. Sorafenib is a multi-kinase inhibitor that has marked clinical benefits in patients with advanced renal cancer and prolonged the mPFS and OS better than sunitinib in clinical trials, especially in Asian populations [8–10]. As a result, sorafenib is still recommended under certain circumstances as the choice for treatment of advanced RCC, especially in Asian patients [8, 11, 12]. The NCCN (National Comprehensive Cancer Network) guidelines recommend sorafenib as treatment of sequential therapy for recurrent or stage IV RCC [10]. Sorafenib targets both the Raf/MEK/ERK signaling pathway and VEGFRs, with antiangiogenic and antiproliferative properties, both of which can inhibit the growth of mRCC. However, treatment resistance still occurs in response to sorafenib. Increasing studies have indicated that activation of the Ras/Raf/MEK/ERK pathways is a major cause of sorafenib failure [10].

TOPK is a member of the MEK family and is highly expressed in many tumor types [13–15]. Bidirectional signal transduction between ERK1/2 and TOPK was demonstrated to promote tumorigenesis, TOPK was reported to be bonded with ERK1/2 and can be activated by ERK1/2 at Thr9 [16, 17]. However, our previous results indicated that T9A-mutated TOPK could still promote tumor proliferation, indicating that other serine or threonine sites in addition to Thr9 might be activated during this process [18].

In this study, we identified Ser32 as a novel site on TOPK that can be phosphorylated by ERK2. We also verified that TOPK is activated at Ser32 during the development of sorafenib resistance and revealed the possible molecular mechanism in advanced RCC. We also explored sorafenib combined with a TOPK inhibitor as a treatment strategy for sorafenib-resistant mRCC. These investigations will be beneficial for the increasing number of sorafenib-resistant patients when the drug is used as a treatment for mRCC.

## Results

### TOPK is highly expressed in advanced RCC

**Fig. 1:**
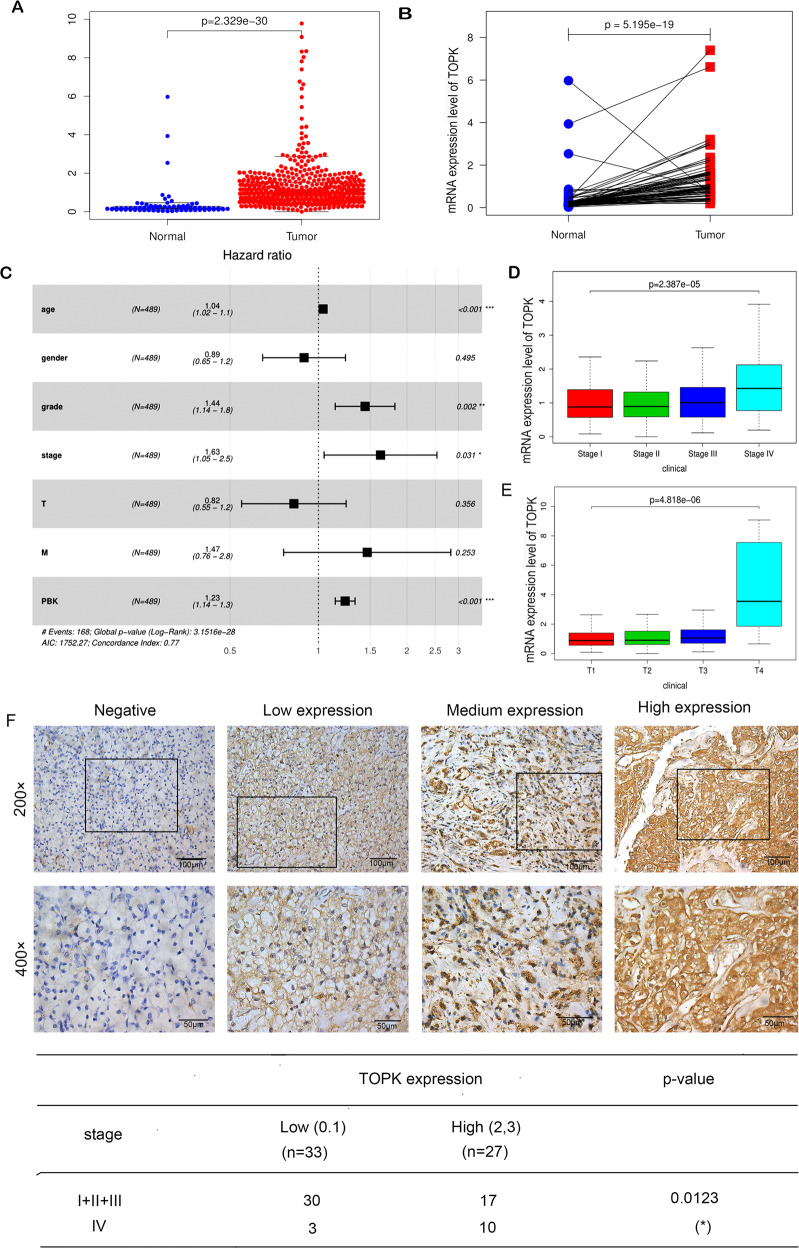
PBK/TOPK is highly expressed in RCC, especially in advanced RCC. Differential expression analysis of PBK/TOPK on RCC datasets of TCGA, the analysis was performed by R project. **A** Differential expression analysis of TOPK between RCC and normal kidney tissue. **B** The paired differential gene expression between normal and RCC tumor tissue. **C** The forest plot of PBK/TOPK expression impact on kidney cancer was analyzed. **D**, **E** The correlation between the expression of PBK/TOPK and classification or stage of RCC was analyzed. **F** The expression of PBK/TOPK in tissues of RCC was determined by IHC. Representative views of IHC staining of PBK/TOPK in patients with different grades of RCC were presented (×400 down; ×200 up), Scale bar, 100 μm or 50 μm. The correlation between the expression of PBK/TOPK and classification and stage of RCC was analyzed.

Kidney cancer datasets from The Cancer Genome Atlas (TCGA) database (https://portal.gdc.cancer.gov/) were utilized to analyze the correlation between TOPK expression and clinical stage, as well as the pathological grade of renal carcinoma and the relationship between expression and overall survival (OS) of patients. All analyses were performed using the R project. The scatter plot of TOPK expression in RCC compared to that in normal kidneys (Fig. 1A) and the paired differential gene expression (Fig. 1B) revealed that TOPK is significantly upregulated in RCC tissues compared to normal kidneys. The forest plot indicated that PBK/TOPK expression was remarkably associated with RCC (*p* < 0.001) and can be used as an independent prognostic factor in kidney cancer (Fig. 1C). Boxplots revealed that expression of PBK/TOPK was strongly associated with T4 classification (*p* < 0.001) and stage IV (*p* < 0.001) RCC (Fig. 1D, E). The correlation of TOPK expression and the survival periods of patients with RCC was analyzed. The results showed that high expression of TOPK was associated with short survival periods of RCC patients (*p* = 0.041) (Supplementary Fig. S1A). The expression of TOPK in RCC tumor tissues of different grades was evaluated by immunohistochemical staining. The results indicated that high expression of TOPK was significant correlated with RCC of stage IV (*p* = 0.0123) (Fig. 1F, Supplementary Fig. S1B). The expression of TOPK in RCC tumor tissues of different grades was detected by western blot, the results indicated that TOPK was lower expressed in normal renal tissues than that in RCC tumor tissues (Supplementary Fig. S1C). Based on these results, we speculated that TOPK might play an important role in advanced RCC.

### Ser32 is a new amino acid site that can be phosphorylated by ERK2 in vitro

**Fig. 2:**
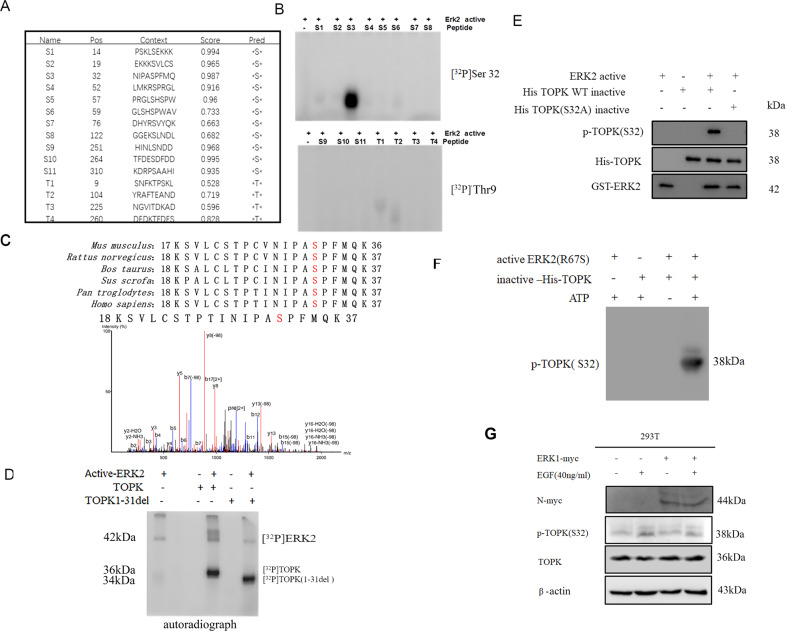
ERK2 phosphorylate TOPK at Serine 32. **A** Potential phosphorylated serine and threonine sites of TOPK were predicted by NetPhos3.0 software program. **B** ERK2 phosphorylated TOPK at S32 in peptide mapping. Synthesized peptides containing potential serine [11] and threonine [4] sites were used as substrates in an in vitro kinase assay with active ERK2 in the presence of [γ- ^32^P] ATP. The results were visualized by autoradiography. **C** The production was obtained from the inactive HIS- TOPK as a substrate of active ERK2 protein by in vitro kinase assay, following the purification and detection of SDS-PAGE, and then the phosphorylation site of TOPK was analyzed by mass spectrometry using Agilent 7100-6545 instrument. **D** The autoradiograph of active ERK2 which phosphorylated inactive total TOPK or segment TOPK (deleted 1-31 amino acids) in the presence of [γ- ^32^P] ATP by in vitro kinase assay. The active ERK2 phosphorylate TOPK at S32 detected by anti-phospho-TOPK (S32) (p-TOPK (S32)) in vitro kinase assay. **E** Wild type His-TOPK (WT) and single mutant His-TOPK (S32A) was used as substrate of active ERK2. Reactive products were determined by Western blot with anti-p-TOPK (S32). **F** Wild type His-TOPK (WT) and mutant His-TOPK (S32A) were used as substrates of autophosphorylation ERK2_R67S in the presence or absence of ATP, and the production was determined by western blot with anti-p-TOPK (S32). **G** pCMV-ERK1-myc or pCMV was transiently transfected into 293T cells. Then the cells were harvested after stimulation with or without EGF and p-TOPK (S32) was determined by western-blotting.

Based on these results, a prediction of potential serine/threonine phosphorylation sites was conducted using NetPhos3.0, with the highest scores (0.994 and 0.987) found on serine 13 and serine 32, respectively, among the fifteen residues (Fig. 2A). All fifteen high-score peptides were designed and synthesized commercially (S1-S11, T1-T4) (PepTide 2.0, Houston, TX, USA). The peptides were incubated with active ERK2 in the presence of [γ-^32^P] ATP in a kinase assay in vitro. Results indicated that peptide S3 (include Ser32) was phosphorylated by active ERK2 more strongly than any other residue (Fig. 2B).

To verify the above results, a TOPK plasmid was constructed with TOPK 1-31 deleted, where the TOPK protein was expressed and purified from bacteria, and a kinase assay in vitro was performed. Results revealed that TOPK was still activated by ERK2 even in the absence of the T9 residue (Fig. 2D). Inactive wild type (His-TOPK-WT) and mutated (TOPK S32A) TOPK proteins were expressed and purified followed by reaction with the purchased active ERK2 kinase or autophosphorylation ERK2_R67S protein respectively, the product of in vitro kinase assay was performed mass spectrometry (MS) analysis, the results indicated that Ser 32 was phosphorylated (Fig. 2C). The phosphor-TOPK (Ser32) (p-TOPK (S32)) antibody was prepared and used to determine whether Ser32 of TOPK was phosphorylated. The results indicated that p-TOPK (S32) was detectable when ERK2 activated TOPK and was undetectable when Ser32 was mutated to alanine (Fig. 2E, F). The pCMV or pCMV-ERK1-N-myc vector was transfected into 293T cells, and p-TOPK (S32) was detected by western blot. The results showed that ERK1 did not significantly phosphorylate TOPK at Ser32 (Line 3 compared to line 1), even if the cells were stimulated with EGF (Line 4 compared to line 2) (Fig. 2G). Taken together, ERK2 instead of ERK1 phosphorylates TOPK, and the phosphorylation occurs mainly at the Ser32 residue.

### ERK2 phosphorylates TOPK at S32 and promotes carcinogenesis of RCC

**Fig. 3:**
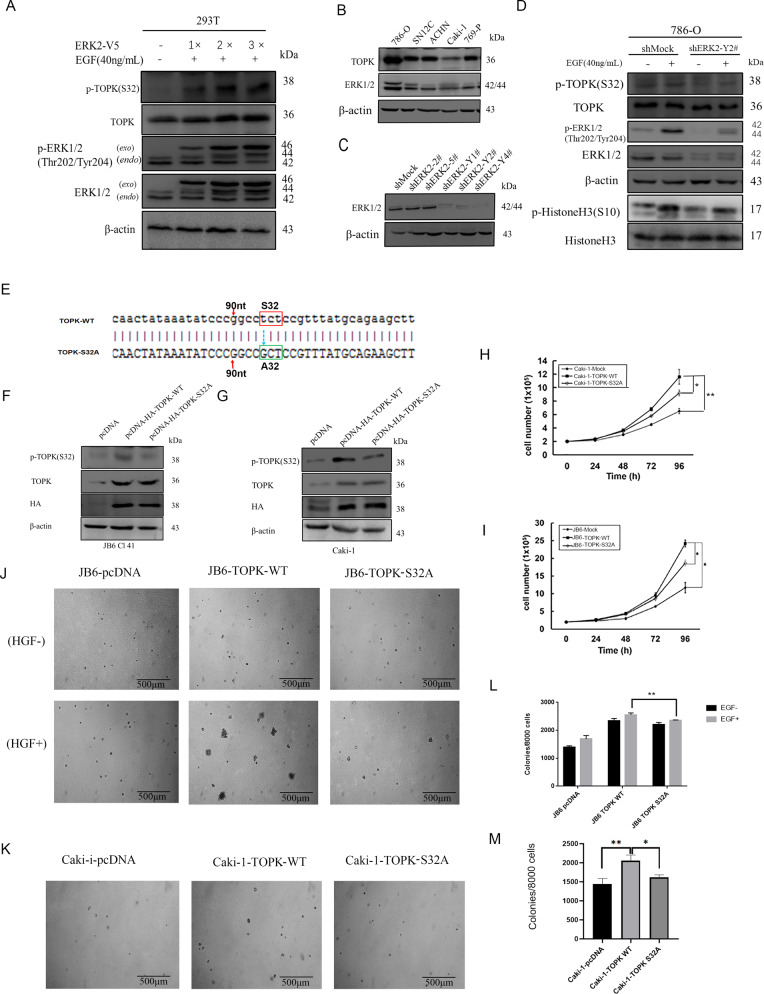
ERK2 effect the phosphorylation of TOPK at S32 in vitro. The phosphorylation of TOPK at S32 promotes carcinogenesis of RCC ex vivo. **A** The phosphorylation of TOPK at Ser32 was detected in 293T cells which were transiently transfected with ERK2 and then activated by EGF at 40 ng/ml, 15 min. **B** TOPK expression level was compared in five Renal cancer cell lines by westen-blot. **C** ERK2 was knocked down in 786-O cell line by lentivirus and the effect of five shERK2 vectors was verified. **D** The expression of p-TOPK (S32), TOPK, ERK1/2 and p-ERK 1/2(Thr202/Tyr204) was detected in these cell lines. **E** The S32 of TOPK was mutant into A32. **F**, **G** pcDNA3, pcDNA3-HA-TOPK-WT and pcDNA3-HA-TOPK-S32A was stable transfected into JB6 Cl41 and Caki-1 cell lines respectively. **H**, **I** The proliferation capability of both cells was visualized by growth curve. **J**–**M** The colony formation in soft agar was performed after the cells were stimulated with or without EGF. Data were represented as means ± SD of triplicate experiments. *, means *P* < 0.05, **, means *P* < 0.01. *exo* mains exogenous ERK2, *endo* mains endogenous ERK2.

The experiments of bonding between TOPK and ERK1/2 was not repeated because such results had been reported. Different quantities of ERK2 plasmid (2 µg, 4 µg, and 6 µg) were transfected into HEK293 cells, and then cells were stimulated with EGF (40 ng/ml). Endogenous phosphorylation of TOPK (S32) was then analyzed by western blotting. Results indicated that increasing the amount of transfected ERK2 plasmid resulted in an increase in endogenous p-TOPK (S32) (Fig. 3A, Supplementary Fig. S3). To investigate ERK2-mediated phosphorylation of TOPK at S32 ex vivo, ERK2 was knocked down in 786-O cells, which highly express TOPK, through lentivirus infection (Fig. 3B–D). The results revealed that levels of p-TOPK (S32) were reduced following ERK2 silencing (Fig. 3D) in RCC cells. The levels of downstream genes of TOPK, such as histone 3 (H3), were also reduced (Fig. 3D).

To investigate the effect of p-TOPK (S32) on carcinogenesis, wild type TOPK (TOPK-WT) was mutated to TOPK-S32A. The wild type or mutant TOPK were overexpressed in the JB6 Cl41 (JB6 for short) and Caki-1 cell lines respectively (Fig. 3E–G) . JB6 is a sensitive mouse epidermal cell line, Caki-1 is a renal cancer cell line that has a low level of expression of endogenous TOPK, and pcDNA3 Mock was used as a control and set up as a stable cell line (Fig. 3F, G). Growth curves and anchorage-independent colony formation of the stable cell lines were analyzed. The growth curve revealed the same tendency in both JB6 and Caki-1 cell lines, with the cells transfected with TOPK-WT growing faster than those transfected with TOPK-S32A, and both cells growing faster than the control cells (Fig. 3H, I). Compared to the JB6-TOPK WT group, the colonies formed by JB6 TOPK S32A cells were significantly fewer in number and smaller in size (Fig. 3J, L). The same results were achieved in Caki-1-TOPK S32A cells (Fig. 3K, M). The above data indicate that mutated TOPK at S32 blocks the growth of tumors ex vivo, suggesting that phosphorylation of TOPK by ERK2 at S32 promotes carcinogenesis in RCC ex vivo.

### The expression of p-TOPK (S32) in sorafenib resistant RCC cells was higher than that in sensitive cells

**Fig. 4:**
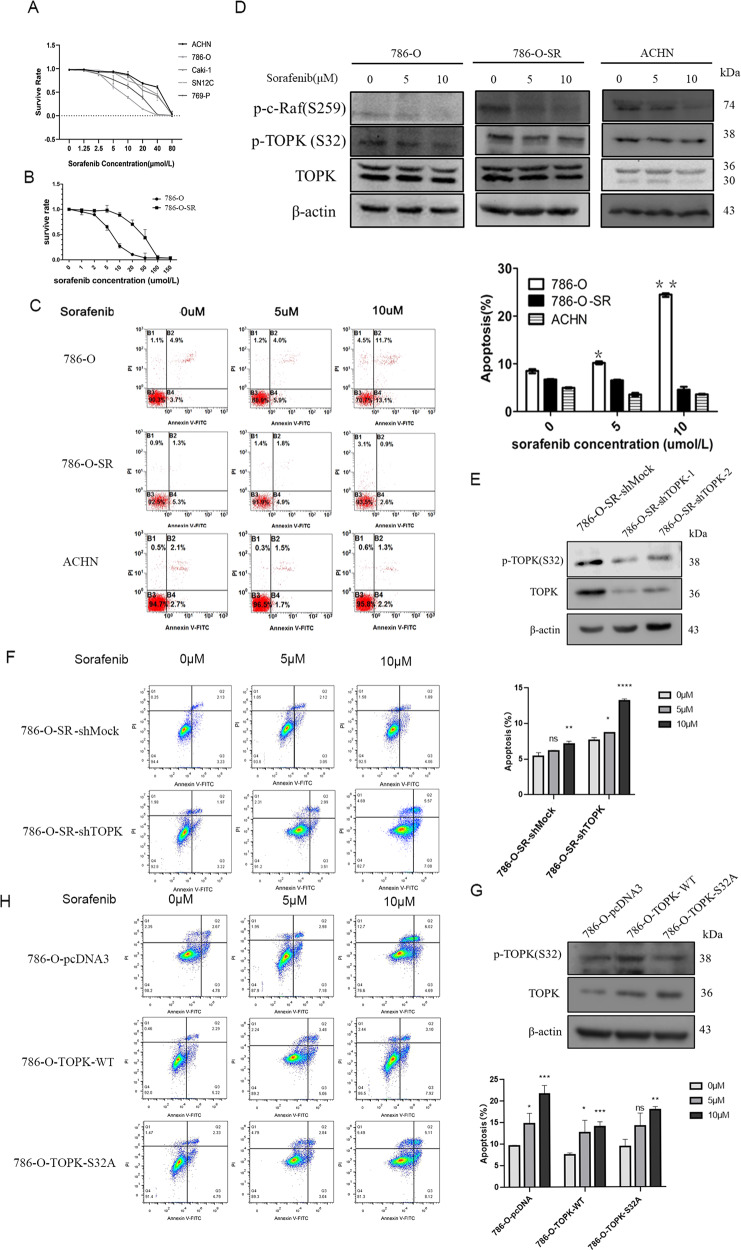
The phosphorylation TOPK S32 was highly expressed in sorafenib resistance RCC cells. **A** The IC50 (half maximal inhibitory concentration) of five RCC cell lines for sorafenib were dectected respectively. **B** The sorafenib sensitive cell line (786-O) was induced to resistance cell line (786-O-SR), the IC50 of the sorafenib in both 786-O and 786-O-SR was detected. **C** The apoptosis of 786-O, 786-O-SR and ACHN in different concentration of sorafenib was detected by flow cytometry, data were represented as means ± SD of triplicate experiments. *, means *P* < 0.05, **, means *P* < 0.01. **D** 786-O, 786-O-SR, ACHN were cultured with media contained with sorafenib of different concentration for 24 h before harvest. The expressions of p-TOPK (S32) and TOPK were determined by western-blot. **E** TOPK was knocked down in 786-O-SR with lentiviral infection. **F** The cells were treated with sorafenib of different concentration (0, 5,10 μM), and flow cytometry was used to determine the cell apoptosis. **G** The plasmids of pcDNA3, pcDNA3-TOPK and pcDNA3-TOPK-S32A were stable transfected into 786-O cell lines respectively. **H** The cells were treated with different concentration of sorafenib (0, 5,10 μM), and flow cytometry assay was used to determine the cell apoptosis.

The resistance of sorafenib in RCC is still an unresolved problem. Here, we further explore whether p-TOPK (S32) is involved in this process. First, the responsiveness of the RCC cell lines to sorafenib was determined by cell viability (%) assays, and results indicated that 786-O and 769-P cells were sensitive to sorafenib, while ACHN and Caki-1 cells were resistant to sorafenib (Fig. 4A). Next, the 786-O cell line was induced to become a sorafenib-resistant cell line (786-O-SR) (Fig. 4B), and the cell viabilities of 786-O and 786-O-SR cells were determined by MTT and flow cytometry. The IC50 of sorafenib for 786-O-SR cells was 15 µM, and that for 786-O cells was 7.5 µM. The flow cytometry results showed that with increasing concentrations of sorafenib, the survival rate of the 786-O-SR cell line was significantly higher than that of the 786-O cell line. Similar results were observed in ACHN cells compared to 786-O cells (Fig. 4C). These results suggest that both 786-O-SR and ACHN cells are sorafenib resistant.

786-O, 786-O-SR and ACHN cells were cultured and incubated with different concentrations of sorafenib, and expression of p-TOPK (S32) and p-c-Raf were determined by western blot. We found that the expression of p-TOPK (S32) in the 786-O cell line was inhibited by sorafenib in a concentration-dependent manner (Fig. 4D, left panel). However, the increasing concentration of sorafenib did not diminished the expression of p-TOPK (S32) obviously in the 786-O-SR and ACHN cell lines (Fig. 4D, middle and right panel). Apoptosis in 786-O cell line increased with higher concentration of sorafenib, as shown in the results of flow cytometry, however, apoptosis in 786-O cell line remained constant in the 786-O-SR and ACHN cell lines, even in the presence of high concentration of sorafenib (Fig. 4C). The expression of p-c-Raf indicated the effective of sorafenib. These results indicate that high expression of p-TOPK (S32) is relevant for sorafenib resistance in RCC.

TOPK was stably knocked down in 786-O-SR and ACHN cells with lentiviral infection (Fig. 4E, Supplementary Fig. S4C). The results of flow cytometry and MTT assay indicated that 786-O-SR-shTOPK and ACHN-shTOPK cells reduced the ability of the tolerance to sorafenib compared with the control groups (Fig. 4E, F and Supplementary Fig. S4B, D).

pcDNA3, pcDNA3-TOPK and pcDNA3-TOPK-S32A were overexpressed in 786-O cell line respectively (Fig. 4G). After treatment with sorafenib, the results of apoptosis and cell viability assay indicated that sorafenib sensitive cells overexpressing TOPK showed increased tolerance to sorafenib compared with the control group, but this trend was rescued in cells after transfected with mutated TOPK at S32 (Fig. 4H and Supplementary Fig. S4A).

### Sorafenib combined with a TOPK inhibitor enhances the sensitivity of sorafenib-resistant RCC cells in vitro

**Fig. 5:**
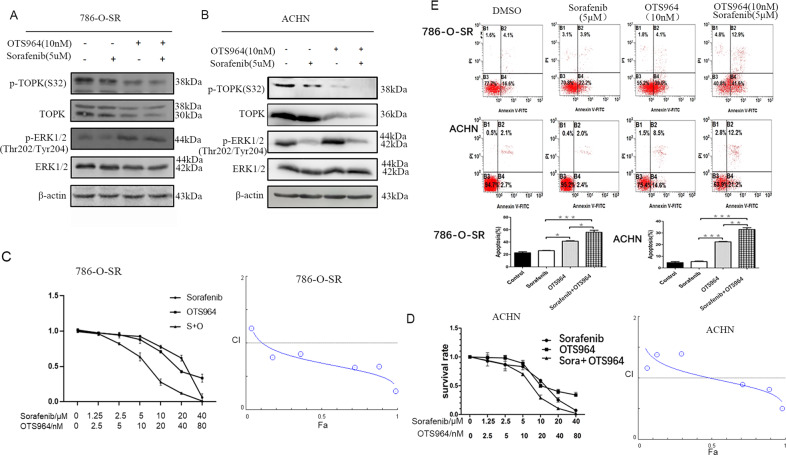
The combination of sorafenib and TOPK inhibitor could promote the apoptosis of the sorafenib resistance RCC cells. **A**, **B** The sorafenib resistance RCC cell lines 786-O-SR and ACHN were incubated with sorafenib, OTS964, or a combination of sorafenib and OTS964 for 24 h. p-TOPK (S32), p-ERK1/2 (Thr202/Tyr204) were determined by western-blot after these cells were harvested. **C**, **D** The survival rate of every group was analyzed, and DPI of sorafenib and OTS964 was calculated. **E** The apoptosis of these cells was detected by flow cytometry. Data were represented as means ± SD of triplicate experiments. *, means *P* < 0.05, **, means *P* < 0.01.

Based on the above results, we investigated whether inhibition of TOPK would suppress sorafenib-resistant RCC cells and restore RCC cell sensitivity to sorafenib. OTS964 is a TOPK inhibitor, with better pharmacokinetic profile for in vivo work than others [19–22]. 786-O-SR and ACHN cells were incubated with sorafenib, OTS964, and a combination of sorafenib and OTS964; DMSO was used as a control. Cell viability was compared using CCK-8 and flow cytometry, while expression of TOPK, ERK1/2 were determined by western blot. Results indicated that the combination of OTS964 and sorafenib led to the highest levels of apoptosis and the lowest expression of p-TOPK among all conditions examined (Fig. 5A, B). The survival rate and flow cytometry results revealed that sorafenib combined with OTS964 was more effective than either sorafenib alone or OTS964 in inducing apoptosis of sorafenib-resistant RCC cells (Fig. 5C–E). Values for the coefficient of drug interaction (CDI) for 786-O-SR and ACHN cells were determined by MTT assay and CompuSyn software (ComboSyn, Inc., Paramus,NJ) according to Chou-Talalay method [23]. Results indicated that the two agents had a synergistic effect in inducing apoptosis of sorafenib resistance RCC cells. Different from that in ACHN cells, expression of p-ERK1/2 in 786-O-SR cells was dramatically increased in the presence of OTS964, it was speculated that there is an unknown mechanism behind this phenomenon (Fig. 5A).

### Sorafenib combined with OTS964 suppresses sorafenib-resistant RCC tumors in vivo

**Fig. 6:**
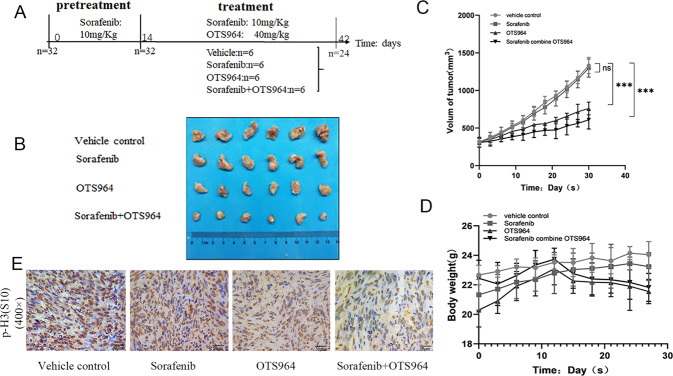
TOPK inhibitor combined with sorafenib suppresses the tumor of sorafenib-resistant RCC. **A** The schedule of animal experiment in the present study was drawn up. **B** The collected tumors were photographed. **C** The grow curves of tumors in different groups were visualized. **D** The bodyweights of mice, ‘**’ (*P* < 0.001) showed a significant difference. **E** Representative IHC images (×400) of phosphorylated H3 (p-H3) in different treatment groups.

A diagram of the in vivo experimental timeline is shown in Fig. 6A. Thirty days after the commencement of treatment, administration of OTS964 significantly reduced tumor size by 20.8%, while the combination therapy resulted in an even higher reduction of 40.5% in tumor size compared to tumor size in the control group. These results were supported by the recorded tumor volume (Fig. 6C) and photographs of the tumors, which were harvested at the end of experiments (Fig. 6B). In addition, the body weights of mice in the three treatment conditions of sorafenib, OTS964, and their combination were not much different from that of the control group, indicating that the toxicity level of the three experimental conditions was not significant (Fig. 6D). The mice treated with the combination of sorafenib and OTS964 were conducted by H&E staining on tissues of liver and kidney. The results supported that the combination of these two drugs did no harm to the organs of the mice (Supplementary Fig. S6). p-Histone H3 (S10) was employed to reflect the expression of phospho-TOPK (S32) in tumor tissues because a phospho-TOPK (S32) antibody suitable for immunohistochemistry was not successfully obtained, and according to our published study, p-H3 (S10) reflects expression of phosphor-TOPK very well [18]. IHC results indicated that p-histone H3 (S10) was highly expressed in tumor tissues treated with sorafenib and was expressed at low levels in those treated with a combination of OTS964 and sorafenib (Fig. 6E).

### HGF/c-MET contributes to activation of TOPK at Y74/S32, directly bypassing the Ras/Raf/ERK cascade and inducing resistance to TKIs in RCC cells

**Fig. 7:**
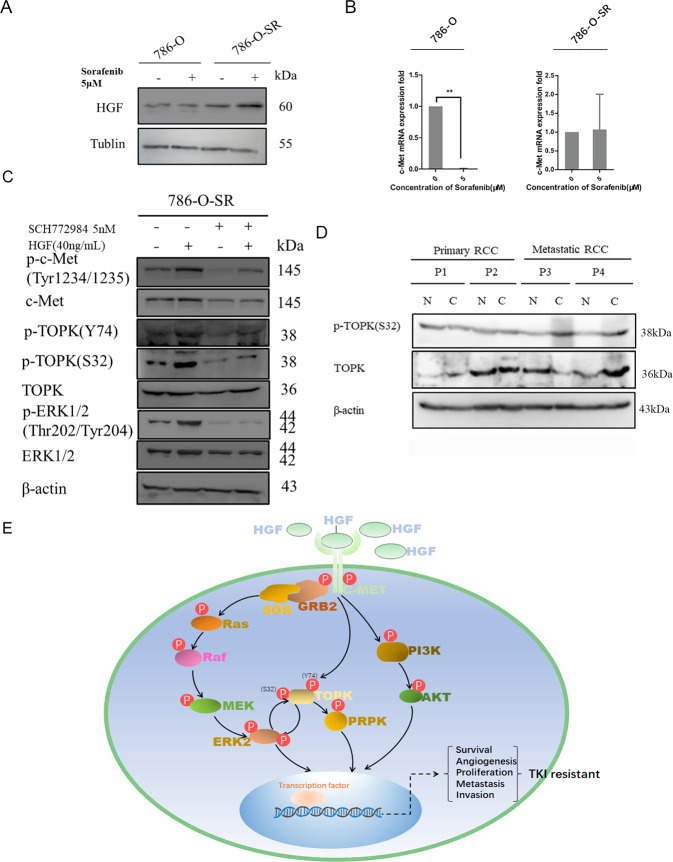
The proposed molecular mechanisms of TOPK’s involvement in sorafenib-resistance RCC. **A** 786-O and 786-O-SR cells were treated with sorafenib (5 uM) or DMSO respectively, and the expression level of HGF protein was analyzed by immunoblotting. **B** 786-O and 786-O-SR cells were treated with sorafenib (7.5 uM) or DMSO respectively, and the mRNA level of c-MET was analyzed by RT-PCR analysis. **C** 786-O-SR cells were treated with SCH772984, and then they were stimulated with or without HGF for 24 h. The expression level of p-TOPK(S32) and p-ERK1/2 (Thr202/Tyr204) were analyzed by western-blot. **D** The expression of TOPK and p-TOPK (S32) in paracancer and RCC tumor tissues was detected in 4 patients (2 cases are primary RCC and 2 cases are mRCC). **E** The flow chart of molecular mechanisms of TOPK’s involvement in sorafenib-resistance RCC was drawn up. Abbreviations and explanations: c-Met, also called hepatocyte growth factor receptor [HGFR], ERK extracellular signaling-regulated kinase, HGF hepatocyte growth factor, MEK Mitogen-Activated Protein Kinase Kinase.

HGF/c-Met was reported to be a driver and biomarker of VEGFR inhibitor resistance in non-small cell lung cancer [24] and to contribute to the proliferation and metastasis of hepatocellular carcinoma (HCC), gastric cancer (GC), etc. To explain the hyperexpression of p-TOPK (S32) in sorafenib-resistant RCC cells, HGF/c-Met was investigated in the present study. First, HGF was detected in both 786-O and 786-O-SR cells. Results indicated that 786-O-SR cells enhanced the expression of HGF compared with their parent 786-O cells (Fig. 7A, 3rd lane compared to 1st lane). The expression of HGF was significantly prompted by Sorafenib in the 786-O-SR cells (Fig. 7A, 3rd lane compared to 4th lane), but hardly changed at all in 786-O cells (Fig. 7A, 1st lane compared to 2nd lane). RT-PCR results indicated that the level of c-MET was restrained remarkably by sorafenib in the 786-O cells compared with that in 786-O-SR cells (Fig. 7B). ERK2 was inhibited in 786-O-SR cells for 48 h using SCH772984, which is a specific inhibitor of ERK1/2. Cells were harvested after incubation with HGF (40 ng/ml) in the media for 30 min, and p-TOPK (S32), p-TOPK (Y74), p-ERK1/2 (Thr202/Tyr204) and p-Met (Tyr1234/1235) were determined by western blotting. Results indicated that p-TOPK (S32) was repressed following inhibition of p-ERK1/2 (Fig. 7C, 3rd lane compared to 1st lane, 4th lane compared to 2nd lane). Our results also showed that SCH772984 can also inhibit a portion of c-MET (Fig. 7C, lane 3 and lane 4 compared to lanes 1 and 2), the inhibited c-MET in turn reduced the level of p-TOPK(Y74). However, the exogenous addition of HGF rescued the downregulation of both p-TOPK (S32) and p-TOPK(Y74) (Fig. 7C, 4th lane compared to 3rd lane). The expression of p-TOPK(S32) in tissues of RCC tumors and the adjacent from four patients were determined by western blot. The results indicated that the level of p-TOPK (S32) in tumors tissue of mRCC patient was significantly higher than that in adjacent tissues, while there was no significant difference in patients with primary RCC (Fig. 7D). A scheme of the possible mechanism that p-TOPK(S32) promotes the sorafenib-resistant in RCC was proposed (Fig. 7E).

## Discussion

Ras/Raf/MEK/ERK pathway is closely related to tumorigenesis and TKI resistance in different types of cancer [25]. TOPK has been reported to be a member of the MEK protein family and is an active form of MEK in cancer tissues [17]. ERK2 has been reported to have proliferative effects, and ERK1 has antiproliferative effects [26, 27]. Zhu et al. demonstrated that ERK2 could bind to and phosphorylate TOPK at T9, which could be associated with the transformation of tumor cells [16]. Herein, for the first time, we reported that Ser32 represents another site of TOPK that can be strongly phosphorylated by ERK2. Phosphorylation of TOPK at S32 is also associated with the transformation and proliferation of RCC cells, and moreover, S32 has a more significant effect on tumor transformation than T9. The clinical correlation analysis based on TCGA indicated that TOPK was markedly increased in T4 classification and stage IV tumors, suggesting that high expression of TOPK is more association with advanced RCC.

Sorafenib is a commonly used multitarget small molecule TKI approved by the Food and Drug Administration [28], but unfortunately, patients receiving sorafenib alone exhibit limited survival benefit due to drug resistance [29, 30]. The mechanism of resistance to sorafenib in RCC is still not fully understood by now [10]. Sorafenib inhibits angiogenesis, resulting in hypoxia inside tumors [31], which in turn increases the production of hepatocyte growth factor (HGF) and expression of c-MET in tumor cells [32–34]. Peters and Adjei reported that sustained sorafenib exposure induces production of HGF, while upregulation of c-Met and p-c-Met in turn activates its downstream factors, including Akt and ERK1/2 [34]. Cascone *et al*. reported that the HGF/c-MET pathway mediates VEGFR inhibitor resistance and vascular remodeling in NSCLC [24]. Our results indicated that HGF and c-MET were expressed at higher levels in sorafenib-resistant cells (786-O-SR) than in sensitive strains (786-O). High level of HGF/c-MET can active TOPK at S32 in sorafenib-resistant RCC cells, even if ERK2 and MEK were inhibited. MET was reported to be bind with and activate TOPK at Y74 in gefitinib-resistant NSCL cancer cells [35], the above results were in consistent with the present study.

Our results confirmed that the positive feedback loop between ERK and TOPK enhances the signaling cascade and plays roles in the development of advanced RCC. The increased level of p-TOPK (S32) during sorafenib treatment indicates the activation of p-c-MET, therefore, p-TOPK (S32) can be employed as a predictive marker of sorafenib resistance in advanced RCC.

Sorafenib resistance remains a major challenge in the treatment of advanced renal cancer, the novel multi-targeted and personalized therapies are worth being further explored. Targeting the MEK/ERK pathway is also not an effective long-term strategy against sorafenib resistance in RCC, because the activated c-MET will eventually lead to cell proliferation and metastasis through other pathway, such as TOPK/ERK, PRPK or PI3K/AKT [24, 34]. It has been reported that targeting COX2/MET/TOPK signaling pathway could promote the apoptosis of gefitinib-resistant NSCLC cells [35]. Our result demonstrated that the combination of sorafenib and TOPK inhibitor could also promote the apoptosis of sorafenib-resistant RCC. Therefore, we speculated that targeting both TOPK and MET could be an alternative therapeutic strategy for TKI-resistant RCC. Till now, none of TOPK inhibitors has been approved for clinical application; however, some existing medicines have been identified as potential substitute, e.g., pantoprazole has been proved to inhibit TOPK effectively [36].

In the current study, we first reported that ERK2 phosphorylates TOPK at Ser32 and promotes tumorigenesis, p-TOPK (S32) is highly expressed in sorafenib-resistant RCC cells. The data suggested that p-TOPK (S32) could serve as a biomarker of the level of benefit sorafenib in advanced RCC patients, the higher of p-TOPK (S32) the less of the benefit. Additionally, we found that the high level of HGF in sorafenib-resistant RCC cells was responsible for activation of TOPK at S32. Targeting TOPK could be an alternative combinational therapeutic strategy to overcome sorafenib resistance in advanced RCCs, facilitating patient selection in precision therapy with sorafenib.

## Materials and methods

### Cell culture

JB6 Cl41, HEK293T, 786-O, ACHN, 769-P, and Caki-1 cells were purchased from ATCC (American Type Culture Collection), SN12C cell line was kindly donated by Dr. Chung Leland W.K. (Cedars-Sinai Medical Center, Los Angeles, USA). All cell lines were cultured following the procedures. To authenticate the cell lines, the short tandem repeat profiling of cells was analyzed, and mycoplasma-negative status of cells was confirmed. Cells were used within 18 passages. All cell lines were passaged and thawed at least three times before they were used for the experiments.

### Induction of sorafenib-resistance in RCC cells

To generate sorafenib-resistant cells, a stepwise increase in the concentration of sorafenib was used to culture 786-O cells, which were verified to be sorafenib-sensitive RCC cells, continuously for 12 months (starting at 5 µM, increasing the concentration by 2.5 µM at each passage, up to a final concentration of 15 μM). The generated sorafenib-resistant RCC cells were designated 786-O-SR cells. The parental cells were cultured without sorafenib and served as controls.

### Antibodies and reagents

The TOPK mouse mAb antibody (sc-293028) was purchased from Santa Cruz Technology, Inc. (Santa Cruz, CA). TOPK rabbit mAb (#4942), phospho-c-Raf (Ser338) (56A6) rabbit mAb(#9427), Raf mouse mAb (#12552), ERK1/2 rabbit mAb (#4695), p-ERK1/2(Thr202/Tyr204) rabbit mAb (#4649), histone H3 (D1H2) XP rabbit mAb (#4499), phospho-histone H3 (Ser10) (D2C8) XP rabbit mAb (#53348), histone H3 rabbit mAb (#4499), MET Rabbit mAb (#8198), p-MET Rabbit mAb (#3077), and HA tag rabbit polyclonal antibody (#3724) were purchased from Cell Signaling Technology (CST), Inc.((Danvers, MA). The β-actin mouse monoclonal antibody (#3700) and secondary antibody against mouse and rabbit were purchased from CST. Anti-HGF antibody (ab178395) was purchased from Abcam, Inc. (Cambridge, MA). The phospho-TOPK at S32 and Y74 antibodies was prepared by Abgent, Inc. (Suzhou, JS). All antibodies were used following the manufacturers’ instructions. Vector plasmids were transfected using Lipofectamine 2000 from Invitrogen, Inc. (Carlsbad, CA). The shRNA constructs against ERK2 were designed by the BioMedical Genomics Center at the University of Minnesota. Sorafenib, OTS964 and SCH772984 were purchased from Selleckchem, Inc. (Houston, TX). G418 and puromycin were purchased from Sigma-Aldrich (St. Louis, MO) and Solarbio, Inc. (Beijing, BJ). EGF and HGF was purchased from R&D Systems, Inc. (Minneapolis, MN).

### Lentiviral infection

Lentiviral expression vectors, including shERK2, vehicle control shMock, pMD2.0G, and psPAX2, were purchased from Sino Biological, Inc. (Beijing, BJ). Viral vector and packaging vectors were transfected into HEK293T cells using Lipofectamine 2000 (Invitrogen, USA) following the manufacturer’s protocols. Six hours later, the medium was replaced, and cells were cultured for 24 h. Viral particles were harvested and infected into RCC cells for 24 h, and then the medium was replaced. Next, cells were cultured for another 48 h and selected with puromycin (1.5 µg/mL) for at least 3 days.

### Western blot analysis

Cells were cultured in 10 cm dishes to 90% confluence and then harvested and lysed with RIPA lysis buffer (lot: R0010, Solarbio, Inc., Beijing, CHN) containing protease inhibitor (MedChemExpress, New Jersey, USA) and phosphatase inhibitor (MedChemExpress, New Jersey, USA). Then, proteins were separated by SDS–PAGE, followed by transfer to polyvinylidene difluoride (PVDF) membranes (Millipore, Billerica, MA, USA). Finally, membranes were blocked and blotted with the corresponding antibodies and detected by enhanced chemiluminescence reagent (Thermo Fisher Scientific, MA, USA) with C300 (Azure Biosystems, CA, USA).

### Anchorage-independent cell transformation assay

Different cell lines were plated in 6-well plates (8 × 10^3^/well) and exposed or not exposed to EGF (20 ng/ml) and cultured in 1 ml of 0.33% BME agar (Eagle basal medium, Sigma-Aldrich Corp.) over 3 ml of 0.5% BME agar containing 10% FBS. Cells were maintained in a 37 °C and 5% CO_2_ incubator for 5–10 days, and then the colonies were counted and scored by microscopy.

### In vivo study

Athymic Balb/c nude mice (six- to eight-week-old female, mean weight is 20 g) were purchased from HFK Bioscience Co., Ltd. Mice were cared for and maintained in the Experimental Animal Center of Xiamen University. Animal experiments were performed following the protocols approved by the Animal Ethics Committee of medical collage of Xiamen University. Mice were housed in a room of specific pathogen-free conditions with a 12 h-day/night cycle with lights on at 8:00 a.m. in a temperature (27 ± 1 °C) and humidity (50 ± 10%) controlled. All mice were allowed free access to water and a balanced diet and were free of all viral, bacterial and parasitic pathogens. All mice were euthanized in their individually ventilated cages with carbon dioxide (25% chamber volume per minute).

786-O-SR cells (5 × 10^6^ cells were suspended in 100 μl PBS) were subcutaneously inoculated into the flanks of mice (a total of 32 mice), and every mouse received daily oral administration of 10 mg/kg sorafenib to maintain sorafenib resistance. Two weeks later, only 24 mouse tumors successfully grew to an average of 300 mm^3^ each. These mice were randomized into four groups by picking random numbers (*n* = 6 per group) and assigned to vehicle, sorafenib, OTS964 or combination therapy groups. Sorafenib and OTS964 were orally administered daily at dosages of 10 mg/kg and 40 mg/kg, respectively, while mice in the control group received oral administration of vehicle. Double blinding was done in this experiment. Tumor volumes (V) were measured every 3 days from their length (l) and width (w), and tumors were harvested 30 days after the commencement of treatments. Tumor volume was calculated using the following formula: V = 0.52 (l × w^2^). Subcutaneous tumors were dissected for further histological examination, and expression of p-histone H3 (S10) in tumor sections was determined by immunohistochemistry.

### Bacterial expression and purification and in vitro kinase assay

pET-His-TOPK-WT and pET-His-TOPK-S32A were expressed in E. coli BL21 bacteria. Isopropyl β-D-thiogalactopyranoside (IPTG) was used to induce protein expression following bacterial growth to an OD600 of 0.6–0.8. Proteins were purified using nickel-nitrilotriacetic acid agarose (Qiagen, Inc., Valencia, CA, USA) and eluted in 200 mM imidazole. Then, proteins were separated by 10% SDS-PAGE. ERK2 active kinase was purchased from Millipore Corp. (Billerica, MA). The reaction system containing active kinase (0.2 μg in a 30 μl reaction), inactive substrate (2 μg) and 1× kinase buffer containing 100 μmol/L unlabeled ATP were incubated at 32 °C for 40 min. Then, samples were resolved by western blot analysis.

### Flow cytometry analysis

Cell apoptosis was determined with the Annexin V–FITC/ PI Detection Kit (Vazyme, Nanjing, JS). Firstly, Cells (2 × 10^5^/well) were seeded in six-well plates and cultured for 12 h followed by treating with sorafenib or OTS964 as designed. Secondly, the cells were digested with trypsin and washed with PBS. Finally, the cells were incubated with Annexin V–FITC plus PI for 15 min and analyzed by FACSCalibur Flow Cytometer (Beckman CytoFlex) and FlowJo VX software.

### Patient clinical samples

Sixty cases of RCC were collected at the initial diagnosis from Xiang’an Hospital of Xiamen University from 2019 to 2021. Samples sizes were chosen to achieve a minimum of triplicates for all experiments. Cohort included RCC patients at stage I, II, III or IV according to TNM staging system. Clinicopathological characteristics for patients and demographic information were summarized in Supplementary Table S1. Immunohistochemically (IHC) stained slides were used to assess the TOPK expression. The low and high TOPK expression in tumor tissues was analyzed on Image J (IHC Profiler) software. Briefly, for each patient sample, we analyzed three different visual fields, and any discrepancies in scores were subsequently reconciled, cut off value with a high level of positivity has diagnostic priority. Intensity score staining was defined as follows: “0”, none; “1”, light; “2”, moderate; and “3”, intense. Then, “0” or “1” was categorized as low expression and “2” or “3” as high expression. Ethical approval was obtained from the medical ethics committee of Xiang’an Hospital of Xiamen University. Written informed consent was obtained from a legally authorized representative for anonymized patient information to be published in this article.

### Immunohistochemistry

Tumor samples were fixed in 4% paraformaldehyde and subsequently embedded in paraffin. Sections of 5 μm were placed on glass slides, and horseradish peroxidase (HRP) staining was performed. After deparaffinization and antigen retrieval, endogenous peroxidases were quenched with H_2_O_2_ and blocked with BSA for 30 min, and sections were incubated with primary antibodies at 4 °C overnight. PBS was used for all dilutions and washes. Slides were counterstained with Mayer’s hematoxylin. Images were obtained at 200× or 400× magnification using a Leica Imaging System Microscope (Leica DM2700).

### Statistical analysis

Statistical analyses were conducted using GraphPad Prism 8 (GraphPad Software Inc., San Diego, CA, USA) or SPSS statistics software version 13 (SPSS Inc., USA). All quantitative data are expressed as the mean values ± SD of at least three independent experiments or samples. Fisher’s exact test two-sided analyze was used to analyze statistically significant differences. The Pearson’s correlation was used to measure the strength of association between two variables. Cox proportional hazards regression analysis was used to analyze the independent factors on the survival prognosis of patients with ccRCC. Differences between groups were analyzed by Student’s *t* test or nonparametric Mann–Whitney *U* test. Continuous variables were expressed as the median (95% confidence interval) or mean ± standard deviation (SD) as appropriate. All in vitro experiments were carried out at least in duplicate, and representative results are shown in the figures. All statistical tests were two sided, and *p* < 0.05 was considered significant (*p < 0.05, **p < 0.01, ***p < 0.001).

### RT-PCR

Total RNA was extracted from cells using an RNA extraction kit from Qiagen Inc. (Qiagen, CA, USA) and converted into cDNA using PrimeScript™ RT Master Mix (Takara). SYBR® Premix Ex Taq™ (Takara) was then used for real-time qPCR. The PCR products were analyzed by Bio-Rad CFX96 Real-time PCR systems (Bio-Rad, CA, USA). Primer sequences for c-MET were as follows: forward: 5’ -CTAG ACACATTTCAATTGGT-3’ and reverse: 5’ -TGTTG CAGGGAAGGAGTGGT-3’, corresponding to nt2262–2625 of human c-Met [GenBank NM_000245.3] and an internal control GAPDH mRNA (forward: 5’ - CACCCATGGCAAATTCCATGGCA-3’ and reverse: 5’ -TCTAGACGGCA GGTCAGGTCCACC-3’. Relative gene expression was calculated using 2 − ΔCT (ΔCT = Ct c-MET − Ct GAPDH).

## Supplementary information

supplementary data file

Table S1

Newly added co-author confirmation letter

aj-checklist

## Data Availability

All data generated or analyzed during this study are included in this article and/or Supplementary Materials. The raw data supporting the conclusions of this manuscript may be requested from the authors.
