# Supplementary material for: TOPK/PBK is phosphorylated by ERK2 at serine 32, promotes tumorigenesis and is involved in sorafenib resistance in RCC
Source: Cell Death Dis. 2022 May 11;13(5):450. doi: 10.1038/s41419-022-04909-3 (PMC9095598; doi:10.1038/s41419-022-04909-3)
Supplement: Supplementary file 1 — supplementary data file [file 41419_2022_4909_MOESM1_ESM.pdf]

Figure legends of supplementary data

- **Fig.S1A. The correlation of TOPK expression and the survival periods of patients with RCC was analyzed .**
- **Fig.S1B**. **TOPK expression on immunohistochemical staining in Fig. 1F was evaluated by imageJ software**, cut off points were showed.
- **Fig.S1C. Expression of TOPK in tissues of normal kidney and different stages of RCC.** Normal renal tissues and RCC tissues at different stages were collected. The cells were lysed with RIPA lysis buffer to extract the total protein, and the expression of TOPK was detected by Western blot.
- **Fig.S3 ERK2 activty TOPK at S32 *in vitro*.** ERK2-V5 or pcDNA3.1 was transient transfected into 293T cells, 48 hours later, cells were lysed after stimulating with EGF (20ng/mL) for 15 min, and protein was extracted, expression of p-ERK1/2 and p-TOPK(S32) were detected by WB respectively.
- **Fig.S4 Overexpress TOPK on 786-O cells or knock down TOPK on ACHN cells effected the ability of sorafenib resistance.** (A) pcDNA3, pcDNA3-TOPK and pcDNA3-TOPK-S32A were stably transfected into 786-O cell line respectively. Then the cells were treated with different concentration of sorafenib (0, 5,10 μM), MTT assay were performed to determine the cell viability. (B, C, D)TOPK was stably knocked down in 786-O-SR and ACHN cells with lentiviral infection. Then the cells were treated with different concentration of sorafenib (0, 5,10 μM), MTT assay were performed to determine the cell viability.
- **Fig.S6. Toxicity of sorafenib combined with OTS964.** Representative images of hematoxylin and eosin (H&E) staining on kidney and liver tissues of the mice， which were treated with combination of sorafenib and OTS964, (original magnification ×20), the scale bar is 200μm. (A) H&E staining on kidney tissue. (B). H&E staining on liver tissue.
- **Table S1 The detail information of clinical samples.**

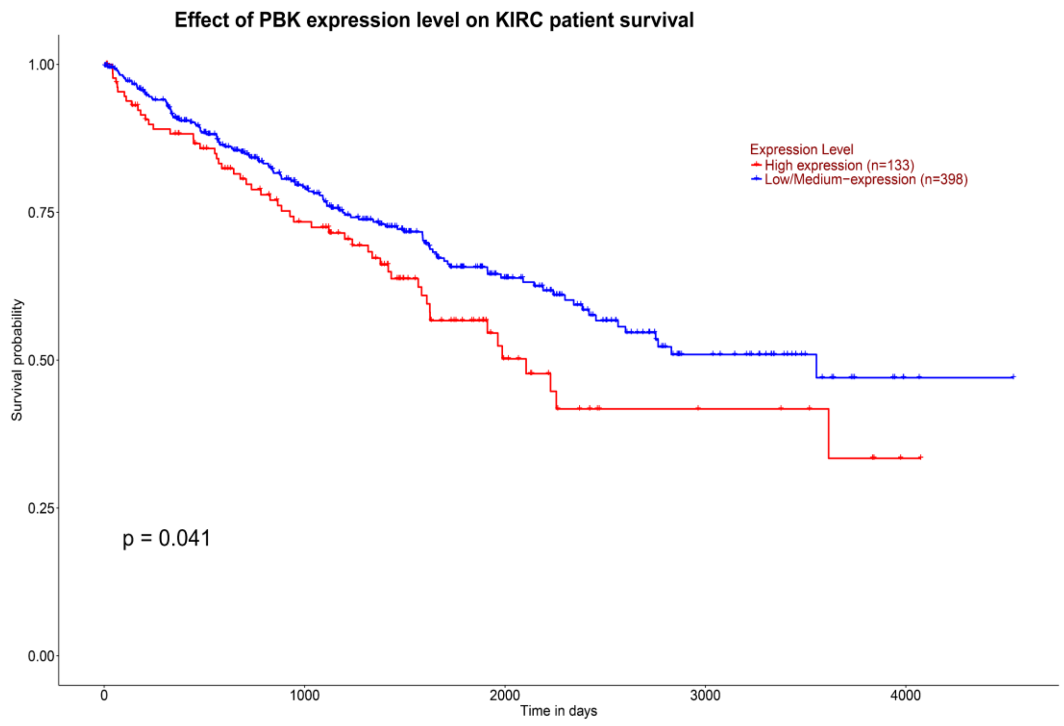

Fig.S1A. The correlation of TOPK expression and the survival periods of patients with RCC was analyzed .

	Sample 1	Sample 2	Sample 3	Sample 4
High Positive（%）	0.007545	1.1273	6.2535	21.9564
Positive（%）	0.1826	14.815	20.2444	38.4659
Low Positive（%）	3.3959	39.1336	27.1763	22.4163
Negative（%）	96.414	44.9241	46.3258	17.1614
Score	Negative	Low Positive	Positive	High Positive

High Positive: High Positive（%）≥10%
Positive: Positive（%）≥20%
Low Positive: Low Positive（%）≥30%
Negative: Negative（%）≥60%
Cut off value with a high level of positivity has diagnostic priority.

Fig.S1B. **TOPK expression on immunohistochemical staining in Fig. 1F was evaluated by imageJ software**, cut off points were showed.

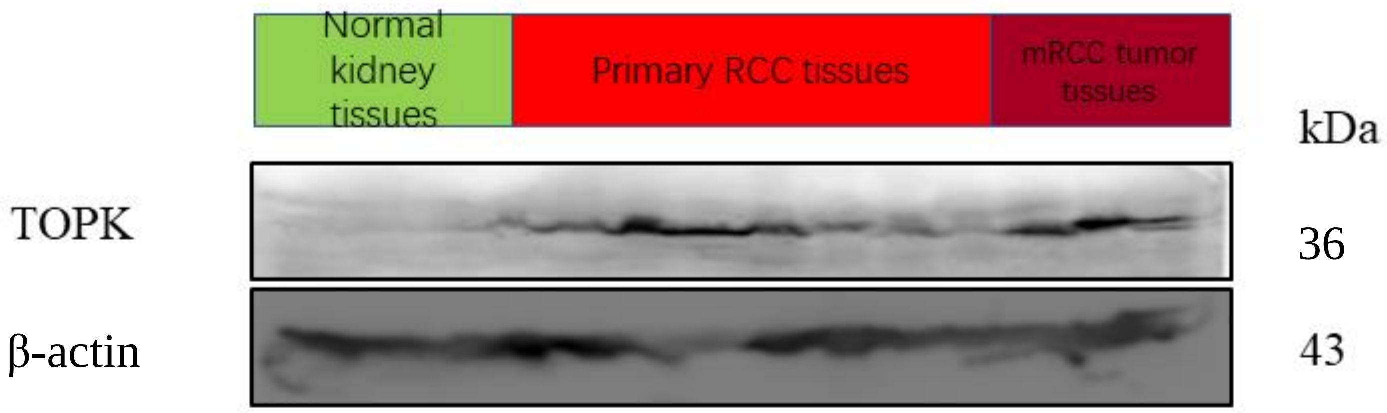

Fig.S1C. Expression of TOPK in tissues of normal kidney and different stages of RCC. Normal renal tissues and RCC tissues at different stages were collected. The cells were lysed with RIPA lysis buffer to extract the total protein, and the expression of TOPK was detected by Western blot.

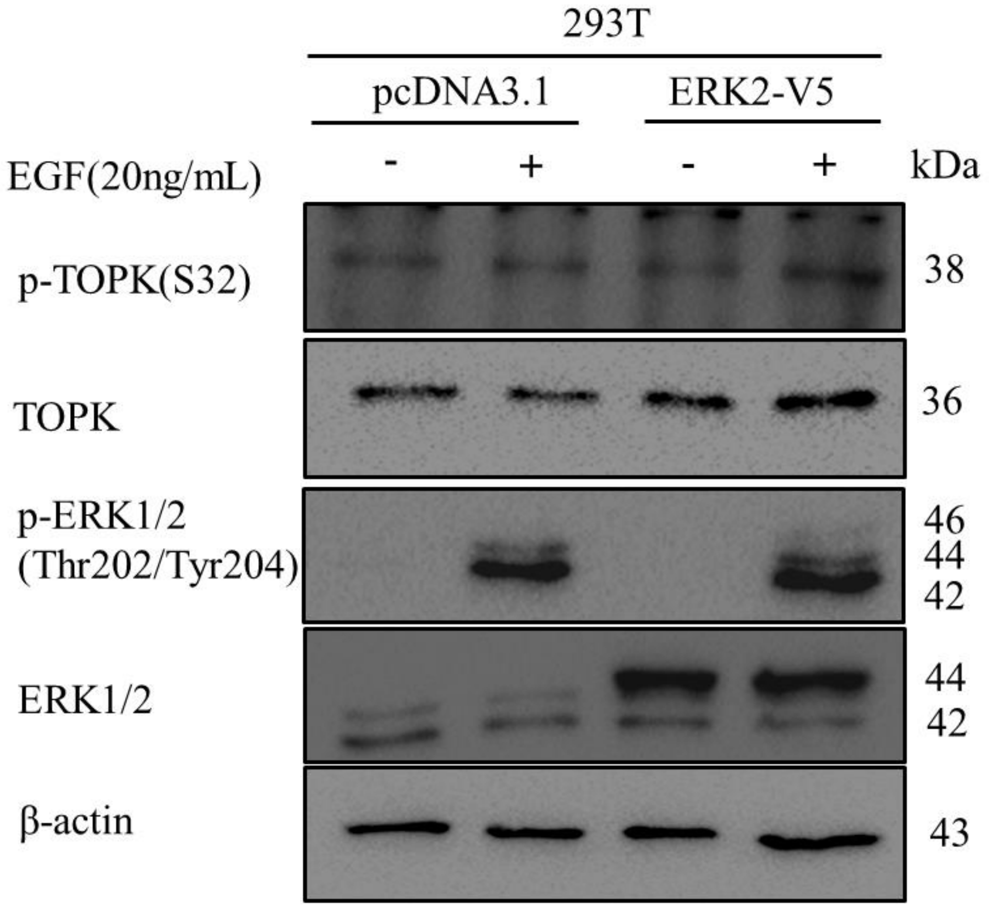

Fig.S3 ERK2 activty TOPK at S32 *in vitro*.

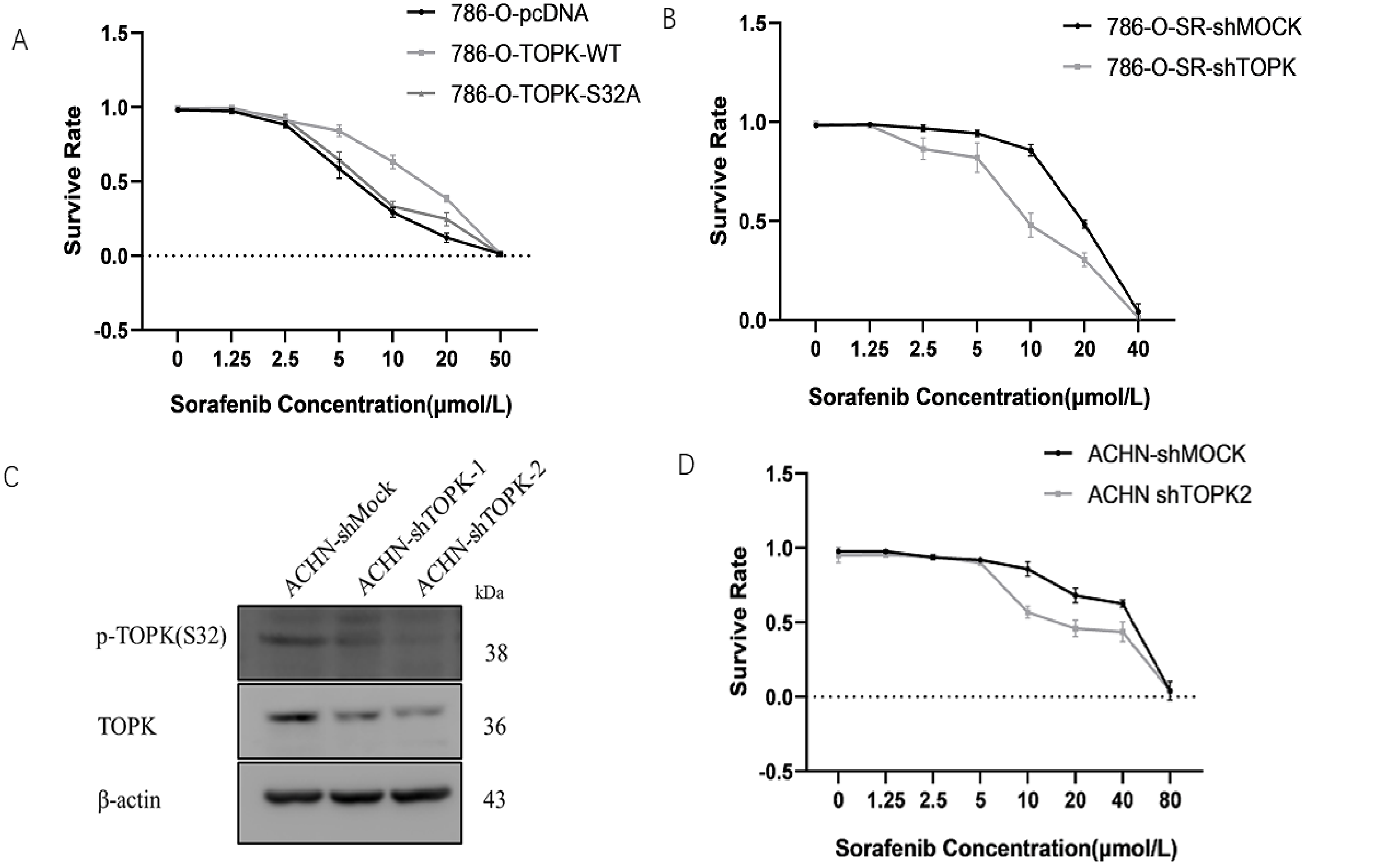

Fig.S4 Overexpress TOPK on 786-O cells or knock down TOPK on ACHN cells effected the ability of sorafenib resistance.

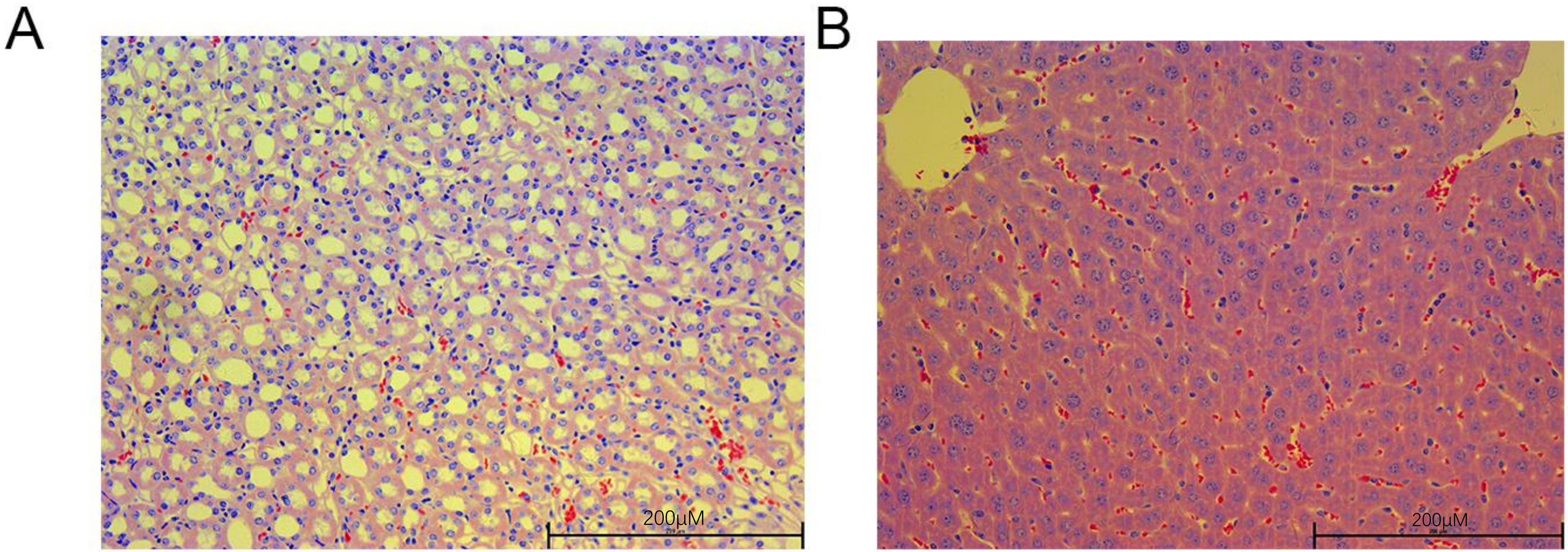

Fig.S6. Toxicity of sorafenib combined with OTS964.
