# Supplementary material for: TOPK/PBK is phosphorylated by ERK2 at serine 32, promotes tumorigenesis and is involved in sorafenib resistance in RCC
Source: Cell Death Dis. 2022 May 11;13(5):450. doi: 10.1038/s41419-022-04909-3 (PMC9095598; doi:10.1038/s41419-022-04909-3)
Supplement: Supplementary file 2 — Table S1 [file 41419_2022_4909_MOESM2_ESM.pdf]

NO.	Gender	Age	diagnosis	Stage	Tumor size	TNM	TOPK expression
1	1	55	1	IV	72 46	T3N0M1	+++
2	1	47	2	IV	61 65	T1N0M1	++
3	1	64	1	IV	39 36	T1NXM1	+
4	2	44	2	III	46 49	T3N0M0	+
5	1	63	2	III	95 81 93	T3N1M0	+
6	2	42	1	II	78 89	T2N0M0	+
7	1	55	1	II	76 80	T2AN0M0	++
8	1	57	1	III	72 101 87	T3AN0M0	+
9	2	68	2	III	29 27	T3CN0M0	++
10	2	70	1	III	56 38 53	T3AN0M0	++
11	2	60	2	III	61 71	T3BN0M0	+
12	1	50	2	II	77 64	T2AN0M0	+
13	1	35	2	III	42 39	T3AN0M0	++
14	2	68	1	II	36 31	T3AN0M0	++
15	2	66	2	II	91 53	T2AN0M0	-
16	1	47	1	III	40 34	T3AN0M0	+
17	2	69	1	III	65 54	T3AN0M0	++
18	1	67	2	II	71 45	T2N0M0	-
19	1	63	2	III	95 81 93	T2AN1M0	+++
20	1	64	2	II	83 65	T2AN0M0	+
21	1	66	2	III	93 77 100	T3AN0M0	-
22	2	21	1	II	133 103	T2BN0M0	-
23	1	42	2	III	35 34	T30N0M0	+++
24	1	40	1	III	50 37	T3AN0M0	+++
25	1	61	2	III	88 67	T3N0M0	-
26	2	60	1	III	71 51	T3CN0M0	+++
27	1	28	2	II	77 58	T2N0M0	+
28	1	48	2	III	70 72	T3AN0M0	++
29	2	51	2	II	98 93	T2AN0M0	+
30	1	64	1	II	117 110 93	T2BN0M0	+
31	1	61	2	II	77.5 73.3 57.	T2AN0M0	++
32	1	67	1	III	121 95	T2N1M0	++
33	1	62	2	II	65 80	T2N0M0	+
34	2	63	1	II	75 57	T2N0M0	++
35	2	46	2	II	75 75	T2N0M0	-
36	2	63	1	II	107 73	T2AN0M0	+
37	1	50	2	II	78 66	T2N0M0	-
38	1	81	2	II	88 56	T2N0M0	+
39	2	52	2	II	77 56	T2N0M0	-
40	2	53	1	II	70 72	T2N0M0	++
41	2	66	1	III	41 43	T2N1M0	+
42	2	47	2	II	99 75	T2N0M0	-
43	1	56	2	III	68 55 72	T3N0M0	++
44	1	55	1	IV	74 46	T3AN0M1	++
45	1	47	2	IV	61 65	T1BN0M1	++
46	1	59	2	IV	46 53	T3ANXM1	++
47	1	64	1	IV	39 36	T1ANXM1	+
48	2	60	1	IV	100 110	T2BN0M1	+++
49	2	68	1	IV	55 46	T1BN1M1	++
50	2	46	2	IV	73 66	T4N0M0	+++
51	1	57	2	IV	57 37	T1BNOM1	+++
52	2	40	2	IV	140 80	T2BN0M1	+++
53	1	68	1	IV	50 41 57	T4N0M0	-
54	2	56	1	III	97 75	T3n0m0	+
55	1	49	1	I	37 44	T1N0M0	-
56	1	51	1	I	35 47	T1N0M0	++
57	2	57	2	I	39 41	T1N0M0	-

58	1	62	1	I	33 40	T1N0M0	+
59	2	63	2	I	39 52	T1N0M0	-
60	2	54	1	I	45 51	T1N0M0	-

Note: In *gender* column: 1 is for male and 2 is for female.

In *diagnosis* column: 1 is for Right kidney, 2 is for Left kidney.
