# Supplementary material for: TOPK/PBK is phosphorylated by ERK2 at serine 32, promotes tumorigenesis and is involved in sorafenib resistance in RCC
Source: Cell Death Dis. 2022 May 11;13(5):450. doi: 10.1038/s41419-022-04909-3 (PMC9095598; doi:10.1038/s41419-022-04909-3)
Supplement: Supplementary file 3 — Newly added co-author confirmation letter [file 41419_2022_4909_MOESM3_ESM.pdf]

Q.D. is the only newly added co-author, the following is the screenshot of her email agreeing to be a co-author.

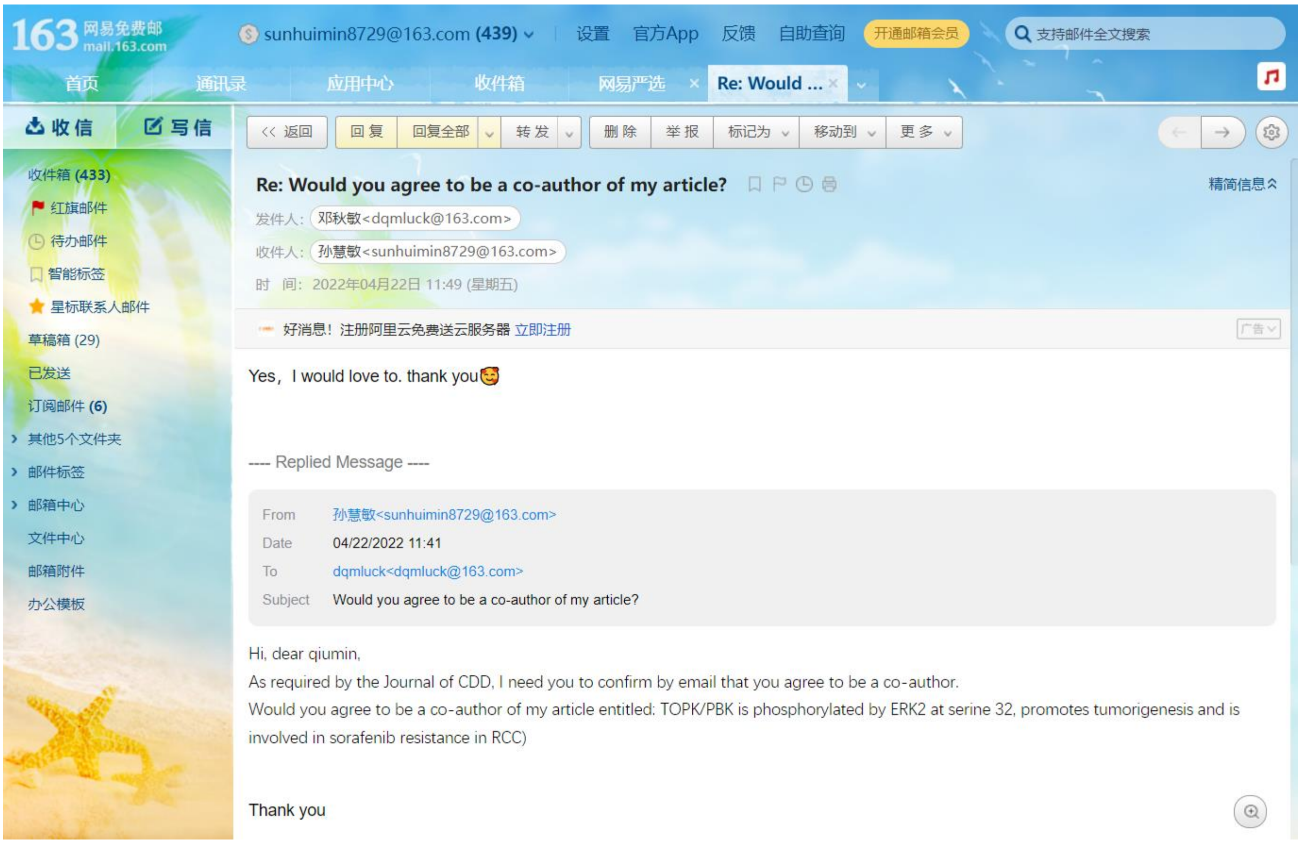

Re: Would you agree to be a co-author of my article?

发件人: 邓秋敏<dqmluck@163.com>

收件人: 孙慧敏<sunhuimin8729@163.com>

时 间: 2022年04月22日 11:49 (星期五)

Yes，I would love to. thank you

---- Replied Message ----

From 孙慧敏<sunhuimin8729@163.com>

Date 04/22/2022 11:41

To dqmluck<dqmluck@163.com>

Subject Would you agree to be a co-author of my article?

Hi, dear qiumin,

As required by the Journal of CDD, I need you to confirm by email that you agree to be a co-author.

Would you agree to be a co-author of my article entitled: TOPK/PBK is phosphorylated by ERK2 at serine 32, promotes tumorigenesis and is involved in sorafenib resistance in RCC)

Thank you
